# The Use of Text Messaging as an Adjunct to Internet-Based Cognitive Behavioral Therapy for Major Depressive Disorder in Youth: Secondary Analysis

**DOI:** 10.2196/40275

**Published:** 2024-05-31

**Authors:** Clarice Walters, David Gratzer, Kevin Dang, Judith Laposa, Yuliya Knyahnytska, Abigail Ortiz, Christina Gonzalez-Torres, Lindsay P Moore, Sheng Chen, Clement Ma, Zafiris Daskalakis, Paul Ritvo

**Affiliations:** 1 Centre for Addiction and Mental Health Toronto, ON Canada; 2 School of Kinesiology and Health Sciences York University Toronto, ON Canada; 3 University of California San Diego San Diego, CA United States; 4 Department of Psychology York University Toronto, ON Canada

**Keywords:** online intervention, randomized controlled trial, major depressive disorder, text message, online, cognitive, behavior therapy, treatment, depression, disorder, symptoms, young adults, wellness, procedure, anxiety, model

## Abstract

**Background:**

As an established treatment for major depressive disorder (MDD), cognitive behavioral therapy (CBT) is now implemented and assessed in internet-based formats that, when combined with smartphone apps, enable secure text messaging. As an adjunct to such internet-based CBT (ICBT) approaches, text messaging has been associated with increased adherence and therapeutic alliance.

**Objective:**

This study analyzed data from the intervention arm of a randomized control trial evaluating 24-week ICBT for MDD (intervention arm) against standard-care psychiatry (waitlist control). The aim of this secondary analysis was to assess MDD symptom improvement in relation to the frequency and content of text messages sent by ICBT participants to Navigator-Coaches during randomized control trial participation. Higher text frequency in general and in 3 conceptual categories (appreciating alliance, alliance building disclosures, and agreement confirmation) was hypothesized to predict larger MDD symptom improvement.

**Methods:**

Participants were young adults (18-30 years) from the Centre for Addiction and Mental Health. The frequencies of categorized texts from 20 ICBT completers were analyzed with respect to MDD symptom improvement using linear regression models. Texts were coded by 2 independent coders and categorized using content analysis. MDD symptoms were measured using the Beck Depression Inventory-II (BDI-II).

**Results:**

Participants sent an average of 136 text messages. Analyses indicated that BDI-II improvement was negatively associated with text messaging frequency in general (β=–0.029, 95% CI –0.11 to 0.048) and in each of the 3 categories: appreciating alliance (β=–0.096, 95% CI –0.80 to 0.61), alliance building disclosures (β=–0.098, 95% CI –0.28 to 0.084), and agreement confirmation (β=–0.076, 95% CI –0.40 to 0.25). Altogether, the effect of text messaging on BDI-II improvement was uniformly negative across statistical models. More text messaging appeared associated with less MDD symptom improvement.

**Conclusions:**

The hypothesized positive associations between conceptually categorized text messages and MDD symptom improvement were not supported in this study. Instead, more text messaging appeared to indicate less treatment benefit. Future studies with larger samples are needed to discern the optimal use of text messaging in ICBT approaches using adjunctive modes of communication.

**Trial Registration:**

Clinical Trials.gov NCT03406052; https://www.clinicaltrials.gov/ct2/show/NCT03406052

## Introduction

### Background

Cognitive behavioral therapy (CBT) is frequently applied in treating major depressive disorder (MDD), with decades of research demonstrating efficacy [1-3]. Recent research has focused on whether technological innovations, particularly internet and phone delivery, can amplify treatment effects, improve adherence, and overcome travel and cost barriers [4-7]. Structured and goal-directed CBT modules are particularly suited to internet-based delivery, and multiple randomized control trials (RCTs) support the efficacy of internet-based CBT (ICBT) in symptom reduction and remission [7-10]. Several RCTs have demonstrated that guided ICBT depression interventions achieve symptom reductions equivalent to in-person CBT [3,11].

### Text Messaging as an Adjunct to CBT

Given positive ICBT effects, particularly in MDD treatment, research has focused on refining technologies to improve engagement and minimize dropout. One focus for improvement is text messaging, the most preferred communication format for young adults, whose mobile phone ownership levels are ≈99% [6,12-14]. Nearly 32% of adolescents and young adults with moderate-to-severe depression report text messaging or video chat in communicating with health care providers [15]. As an adjunct to CBT, text messaging can reduce nonadherence and increase treatment engagement and homework completion [16,17].

### The Efficacy of Text Messaging as an Adjunct to ICBT Treatment for Depression

Text messaging in CBT for depression has been largely aimed at increasing client engagement and therapeutic alliance while minimizing nonadherence. Aguilera et al [18], for example, found that adjunctive text messaging to ICBT for depression was associated with increased attendance and adherence, and reduced depressive symptoms during a 16-week group intervention. While significant between-group differences were not observed, participants in the ICBT plus texting group sustained therapy longer and attended more sessions than clients in the ICBT alone group [18]. Kobak et al [16] examined the effectiveness and user satisfaction of a 12-week technology-enhanced ICBT intervention for depressed youth that used individualized texts constructed by therapists that provided homework reminding and generic support while assisting outcome measurement. A total of 16 clinicians who treated adolescents with elevations on the Quick Inventory of Depressive Symptomatology (score >11) were randomly assigned to (1) in-person treatment as usual (TAU) or (2) TAU in combination with ICBT programming and text messages (ICBT+TAU). Results showed that symptom improvement was significantly greater in the ICBT-TAU plus text-messaging arm than in the TAU controls. Clinicians and participants reported high levels of user satisfaction, with 95% (n=62) of participants reporting “helpful” text exchanges with therapists and 100% (N=65) indicating they would use text messaging again for between-session communications [16]. In a systematic review of the use of text messaging in mental health care, Berrouiguet et al [14] found that RCT data indicated text messaging improved treatment adherence and symptom surveillance. The authors also found that therapy with text-messaging components was associated with increases in appointment attendance and client satisfaction. Agyapong et al [19] sent twice-daily supportive texts to a sample of 54 participants over a 3-month period in a singly-blinded RCT. Participants in the text message arm had significantly lower scores on the Beck Depression Inventory-II (BDI-II) compared to control participants (mean 8.5 [SD 8.0] vs mean 16.7 [SD 10.3]; *P*=.003) [19].

### Study Plan

Despite the efficacy of ICBT interventions for depression, few studies have intensively investigated the intervention mechanisms associated with reduced depressive symptoms [10]. In this secondary analysis, we examined the contents and frequencies of texts sent from participants to Navigator-Coaches (NCs) in relation to depression improvement during a 24-week ICBT intervention for MDD in young adults (18-30 years). A higher frequency of total and alliance-specific texts from participants was hypothesized to predict larger MDD symptom improvement.

## Methods

### Participants

This secondary analysis uses participant data from the intervention arm of a waitlist control, parallel, 2-arm RCT conducted at the Centre for Addiction and Mental Health in Toronto, Canada. The RCT evaluated the efficacy of a web- and telephone-based ICBT intervention for young adults (ages 18-30 years) diagnosed with MDD. The RCT design and primary analysis results [10] have been described elsewhere. All participants were diagnosed by a psychiatrist from the Centre for Addiction and Mental Health, with diagnoses confirmed with a mini international neuropsychiatric interview administered at the final screening visit. Excluded were (1) individuals currently receiving weekly structured psychotherapy; (2) individuals who met the DSM-5 (Diagnostic and Statistical Manual of Mental Disorders) criteria for severe alcohol or substance use disorder in the past 3 months; (3) individuals who demonstrated clinically significant suicidal ideation, defined as imminent intent, or individuals who had attempted suicide in the past 6 months; or (4) individuals diagnosed with borderline personality, bipolar disorder, schizophrenia, or obsessive-compulsive disorder. A total of 45 participants were randomly assigned to the ICBT arm (n=22) or to the standard psychiatric care waitlist control arm (n=23). The 20 ICBT completers were the focus of this study [10].

### Intervention

Both groups received standard psychiatric care, defined as 1 monthly session with a psychiatrist from Centre for Addiction and Mental Health, focused primarily on medication adjustment. Participants in the ICBT intervention group additionally received access to NexJ Connected Wellness (Next Health Inc), a cloud-based digital platform that participants used to securely text NCs, track steps recorded on wearable devices, and access the 24 “workbook” chapters reflecting CBT modules focused on MDD treatment. There were additional telephone sessions with a Health Navigator-Coach (1 hour weekly for 24 weeks for a total of 24 hours) to apply CBT content and support behavior change [10]. Workbook topics included modules entitled Living by Your Truths, Overcoming Wired-ness and Tired-ness, Mindfulness and Relationships, Loss and Grief, and Resilience, Befriending Ourselves, Befriending Your Body With Exercise, Body Image and Mindfulness, Intimacy, Forgiveness, Overcoming Procrastination, Dealing With Negative Moods, Stress Resilience, Overcoming Performance Anxiety, and Cultivating Inspiration [10].

The purpose of the text messages between the participant and NC was to raise and answer questions about ICBT content, reinforce insights on ICBT themes, strengthen the therapeutic alliance, and promote specific behavior changes. NCs used the dialogue exchanged over text messages to structure phone call sessions to meet clients’ needs.

### Coding of Client (Participant) Text Messages

Participant messages were securely transmitted via NexJ Connected Wellness, encrypted, and stored per privacy and security requirements and participant consent. The contents of messages sent from participants to NCs were coded using the procedure identified by Svartvatten et al [20]. All messages from intervention participants (n=20) were grouped via content analysis to determine conceptual categories and develop a comprehensive system that encompassed all client messages. After messages were analyzed, 2 authors (CW and LPM) randomly selected messages from each participant and jointly coded them to ensure the criteria for each category were agreed upon. They then separately coded 3 participants each, to ensure that the categories developed could be used as a reliable template.

The coding categories were as follows:

1. Client greeting NC (alone; ie, no other message following).
2. Client disclosure to update NC (status updates, exchange of status information).
3. Client requesting specific action from NC.
4. Client thanking NC.
5. Client scheduling with NC (organizing, arranging, or coordinating times to communicate with NC, or sending a message regarding planned/attempted communication).
6. Client confirmation or affirmation, agreement (“sounds good”, “that works”).
7. Client requesting information or clarification from NC.
8. Client apologizing to NC.
9. Client indicating actions planned to be undertaken or info they plan to provide to NC.
10. Client expressing appreciation for alliance with NC (Including expressing that they value NC and enjoy communicating with NC).
11. Client novel disclosure to build alliance.
12. Client wishing NC well (have a good day, enjoy your weekend, hope you are well, etc).
13. Client disclosure regarding substance use or abuse.

Based on clinical observations of patients and their engagement in text messaging, a first hypothesis was that 3 specific message types would predict treatment efficacy: client expressing appreciation for alliance with NC (expressing their valuing of the NC and their appreciation of communications with the NC), client confirmation or affirmation-agreement, and alliance building disclosure. A second hypothesis was that participants who sent more messages would experience more symptom reduction after the 24-week intervention due to the observed increased (text message) engagement.

### Measures

The primary outcome measure was the BDI-II [21] while the Beck Anxiety Inventory (BAI) [22], and the Brief Pain Inventory (BPI) [23] were additional measures collected at baseline, 12 weeks, and 24 weeks postrandomization. In this study, we included the BAI and BPI in the analysis because significant within-group and between-group differences were observed in both measures among the original RCT results. Accordingly, we were curious about whether these observed improvements in functioning were significantly associated with the text message intervention component.

### Statistical Methods

Statistical analyses were performed using the SAS Enterprise Guide (version 7.1; SAS Institute). All 2-sided *P* values <.05 were considered statistically significant.

Descriptive statistics summarized participant baseline characteristics while means and SDs summarized continuous measures, and frequencies and proportions summarized categorical measures. The BDI-II change score was calculated as baseline BDI – postintervention BDI, such that higher BDI-II change scores represented greater reductions in depression symptoms. Univariate linear regression was used to test the association between the number of texts sent per category and the BDI change score. Multivariable linear regression was used to further test these associations, adjusting for participant sex, age, BPI, BAI, and total texts sent and texts sent per category. Backward variable selection was used to determine the most parsimonious model. A covariate required a *P* value <.1 to stay in the final model. Regression diagnostics were assessed to verify model fit. Any influential observations with a Cook distance >0.2 were removed from the model.

### Sample Size Considerations

This study is a secondary analysis of a previously published RCT [10,24] that originally aimed to enroll 168 participants, with 50% (n=84) of the participants from a First Nations background and the other 50% (n=84) from all other ethnic backgrounds, stratified into 2 intervention groups and 2 waitlist control groups (ie, 42 per group). However, participant enrollment was reduced due to the reluctance of individuals from First Nations backgrounds to participate, despite extensive recruitment efforts. Additionally, given that each recruited patient had to undergo an extensive psychiatric exam to establish an MDD diagnosis, we confronted a limit to the pace of psychiatric examinations that could be scheduled given the existing staff of psychiatrists.

As this is a secondary analysis, no formal power calculations were performed. This approach adheres to the International Council for Harmonization E9 statistical principles for clinical trials that state sample size should be “determined by the primary objective of the trial” [25]. Our study focuses on estimating the effect sizes and corresponding 95% CIs. Findings from this study will need to be confirmed in a larger, independent study.

### Ethical Considerations

Ethics approval was obtained from the Research and Ethics Boards of the Centre for Addiction and Mental Health (Protocol Reference #115/2016-01) and York University (Certificate #2017–154) in Toronto, Canada (Clinical Trial registration.gov NCT03406052). All participants provided in-person written consent for the use of data in primary and secondary analyses. Participant confidentiality was maintained throughout the study via careful deidentification of data [10,24].

## Results

**Figure 1:**
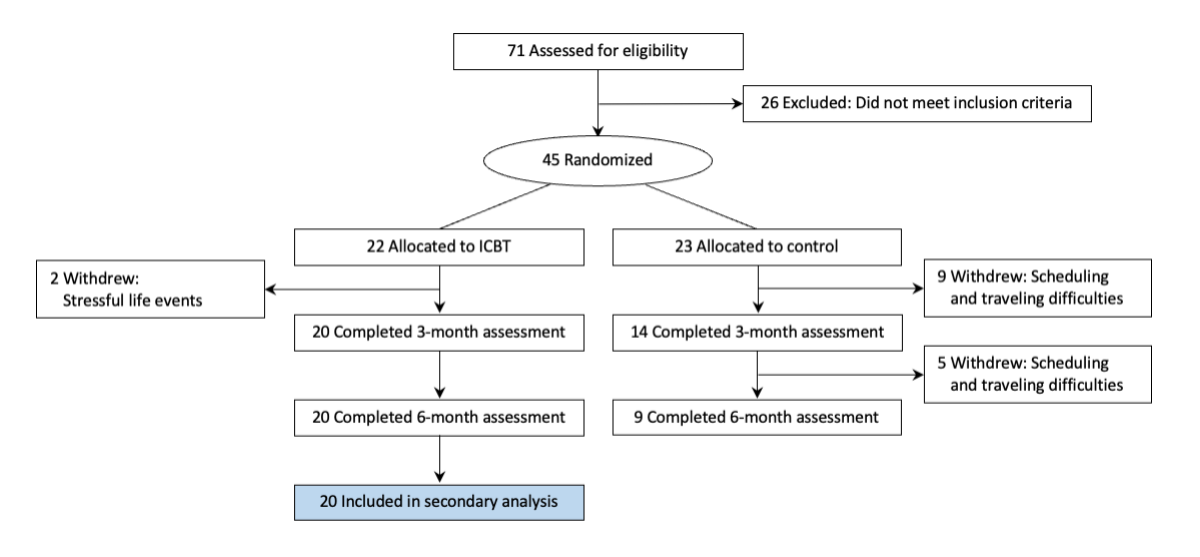
CONSORT flow diagram. ICBT: internet-delivered cognitive behavioral therapy.

In total, 22 participants recruited from February 2018 to September 2018 were enrolled in the ICBT intervention. Of the 22 ICBT participants, 2 withdrew due to stressful life events. This secondary analysis is based on the 20 ICBT intervention completers. Figure 1 summarizes the flow of participants.

The sample of 20 participants was composed of 11 women and 9 men (18 to 30 years) whose mean age was 24.3 (SD 3.4) years. All were psychiatrically diagnosed with MDD and had completed a 24-week web-based intervention aimed at reducing depression symptoms. All were registered outpatients at the Centre for Addiction and Mental Health in Toronto. A BDI-II >14 was required for study eligibility. Their baseline characteristics (including BPI and BAI scores) are presented in Table 1.

**Figure 2:**
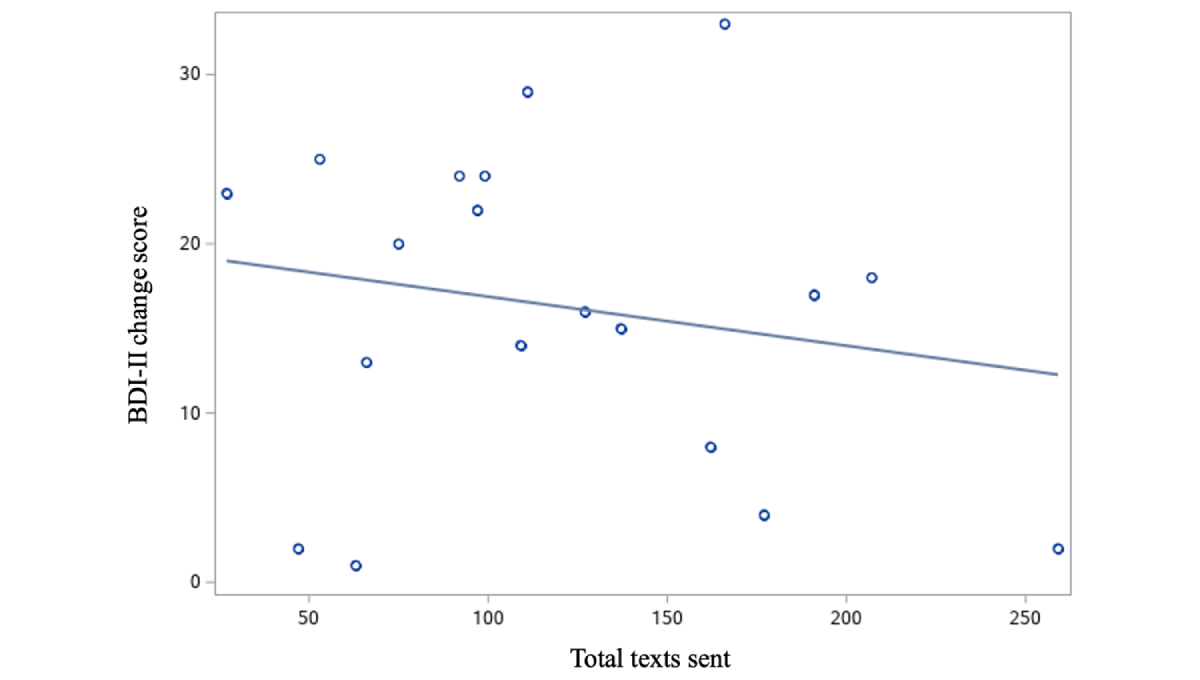
Visualization of the relationship between total texts sent and BDI-II Change (n=19). r(17)=–.188, *P*=.44. BDI-II: Beck Depression Inventory-II.

The BDI-II outcome change (improvement) scores (baseline minus final) ranged from 1 to 33 points, with a mean of 15.60 (SD 9.75). Univariate linear regression was used to test associations between BDI-II improvement and demographic and text messaging variables, with results presented in Table 2. Regression results showed that total texts sent were negatively associated with BDI-II improvement (β=–0.029, 95% CI –0.11 to 0.048). A visualization of this relationship is presented in Figure 2. Text messages related to agreement confirmation and alliance building disclosure were also negatively associated with BDI-II improvement, while text messages related to appreciating alliance were positively associated with BDI-II improvement. Women showed greater BDI-II improvement than men. Age was negatively associated with BDI-II improvement, whereas baseline BPI, and baseline BAI were positively associated with BDI-II improvement.

Multivariable linear regression was used to test associations between BDI-II change and the 3 text message categories, controlling for selected confounders (see Table 3). BDI-II improvement was negatively associated with appreciating alliance texts in model 1 (β=–0.096, 95% CI –0.80 to 0.61), alliance building texts in model 2 (β=–0.098, 95% CI –0.28 to 0.084), and agreement confirmation texts (β=–0.076, 95% CI –0.40 to 0.25) and total texts in model 3 (β=–0.047, 95% CI –0.10 to 0.006).

Altogether, these results show that the adjusted effect of text messaging on BDI-II improvement was uniformly negative across all 3 models—that is, more text messaging, in general, and in all 3 categories, was associated with less depression improvement.

The effect of sex (being female), controlling for covariates, was associated with large BDI-II improvements in all 3 models (β_s_=9.55-10.60).

**Table 1:** Baseline characteristics and content and frequency of text messages of 20 ICBT^a^ participants.

			Variables, mean (SD)
**Baseline characteristic**			
	Age at enrollment in years	24.3 (3.4)	
	Female, n (%)	11 (55)	
	BDI-II score^b^	29.2 (8.22)	
	BPI score^c^	2.95 (2.52)	
	BAI score^d^	28.15 (8.22)	
**Text messages by content**			
	Agreement confirmation	25.60 (15.85)	
	Alliance building disclosure	46.20 (71.35)	
	Appreciating alliance	8.80 (11.47)	
	Total texts sent	135.90 (95.65)	

^a^ICBT: Internet-based cognitive behavioral therapy.

^b^BDI-II: Beck Depression Inventory-II.

^c^BPI: Brief Pain Inventory.

^d^BAI: Beck Anxiety Inventory.

**Table 2:** Univariate linear regression models testing associations between BDI-II^a^ change and demographic and text messaging variables^b^.

Model	Total, n	β (95% CI)	*P* value
Sex (female vs male)	20	6.95 (–1.86 to 15.76)	.12
Age	20	–0.86 (–2.21 to 0.50)	.20
Baseline BPI^c^	20	0.97 (–0.88 to 2.82)	.28
Baseline BAI^d^	20	0.095 (–0.49 to 0.68)	.74
Total text sent	19	–0.029 (–0.11 to 0.048)	.44
Agreement confirmation	20	–0.13 (–0.42 to 0.17)	.39
Alliance building disclosure	19	–0.021 (–0.21 to 0.17)	.82
Appreciating alliance	18	0.21 (–0.46 to 0.88)	.52

^a^BDI-II: Beck Depression Inventory-II.

^b^N across models vary due to the exclusion of influential cases (Cook D >0.20).

^c^BPI: Brief Pain Inventory.

^d^BAI: Beck Anxiety Inventory.

**Table 3:** Multivariable linear regression models testing the associations between text categories and BDI-II^a^ change, adjusted for selected confounders^b^.

Model and predictor			Adjusted β (95% CI)		Adjusted *P* value
**1^c^ (N=18)**					
	Appreciating alliance messages	–0.096 (–0.80 to 0.61)		.78	
	Sex (female vs male)	9.55 (–0.81 to 19.9)		.07	
**2^d^ (N=19)**					
	Alliance building disclosure messages	–0.098 (–0.28 to 0.084)		.27	
	Sex (female vs male)	10.4 (1.46 to 19.3)		.03	
**3^e^ (N=20)**					
	Agreement confirmation messages	–0.076 (–0.40 to 0.25)		.63	
	Total text messages sent	–0.047 (–0.10 to 0.006)		.08	
	Sex (female vs male)	10.6 (2.10 to 19.1)		.02	

^a^BDI-II: Beck Depression Inventory-II.

^b^N across models vary due to the exclusion of influential cases (Cook D >0.20).

^c^*R*^2^=.23, *F*_2,15_=2.19, *P*=.15.

^d^*R*^2^=.28, *F*_2,16_=3.07, *P*=.07.

^e^*R*^2^=.40, *F*_3,16_=3.53, *P*=.04.

## Discussion

The purpose of this secondary analysis was to assess the relationship between depression reduction and the content of texts sent by ICBT participants to NCs during RCT participation [10]. Texts were coded by 2 independent coders and associations between text categories and treatment benefits were assessed using multi-variable linear regression.

To predict better treatment effects, 3 categories were hypothesized: appreciating alliance, alliance building disclosures, and agreement confirmation. Although predicted to be positive, results indicated that the effects of text message frequency on BDI improvements across categories were uniformly negative. A negative association was also observed between the total texts sent and BDI-II improvement. These findings suggest that when participants engaged in more text messaging, whether in identified categories or generically, there were no additional benefits or benefits were reduced.

It is important to place these results in the context of a study where 60% (n=12) of intervention participants achieved remission (BDI-II<14) [10]. Because the ICBT intervention was multi-modal, positive change may have been due to other intervention components besides text messaging. It is even possible that some texts were sent because of doubts or anxieties about the therapeutic alliance rather than perceptions of strong alliances. In any case, trial-related benefits, although hypothesized as linked to text message exchanges, were apparently derived from other modes of multi-modal intervention.

In comparing this study with the literature on text-messaging adjuncts to CBT, multiple findings align with our results regarding the absence of statistical associations between text message activity, and reduced symptomatology. Aguilera et al [18] found in their clinical trial of automated text messaging (adjunctive to CBT for depression), that participants who actively texted did not show statistically significant symptom reductions when compared to controls. The authors remarked that they did not hypothesize direct significant associations because the primary texting function was to reduce attrition and promote treatment engagement [18]. Furber et al [26] found that participants enrolled in a telephone-based psychotherapy intervention with an individually-tailored text adjunct did not show significant between-group differences in clinical outcomes versus controls (ie, phone-based psychotherapy without texting), although both groups showed significant symptom improvements postintervention.

It is possible that text use is helpful at moderate frequencies whereas higher frequencies can exacerbate depressive symptoms, hindering coping skill development [27]. Studies on participant satisfaction, engagement, and symptom reduction in association with psychotherapy-adjunctive text messaging suggest participants who reported satisfying texting indicated optimal exchange frequencies of 1-2 daily [28]. Additional studies have indicated associations between problematic smartphone use and poor sleep quality, depression, and anxiety, suggesting that despite positive intentions, excess text use can result in less engagement with nondigital living [29,30]. In particular, research has documented problematic use thresholds where more text messaging is associated with fewer initiated calls and disengagement from the communication skills applied in voice-based, in-person interactions [31,32]. For example, Tamura et al [33] defined excess smartphone use (for social network services and web-based chat) as >2 hours of use daily, an amount associated with higher depression risks. Our results do not suggest that texting had negative results therapeutically but that more frequent use was associated with less benefit.

Our study differed from other text messaging studies (adjunctive to counseling) because the text message content was not predetermined by central design principles [12]. Instead, message structures evolved from the therapeutic dialogue undertaken between participant and coach, and were linked to and facilitated by other intervention components, for example, weekly phone counseling (1 hour/call), participant interactions with 24 web-based workbooks and 56 videos, and the use of Fitbit wearables in pursuit of walking goals [10]. The messages sent by participants were the focus of the qualitative and quantitative analyses undertaken, emphasizing the types of messages participants created that reflected their participation in therapeutic dialogue.

Other methods have been aimed at discerning tools that result in optimal text message formats aimed, for example, in influencing engagement with an internet-based smoking cessation program [12]. The 4 design features tested in the above study were (1) personalization (incorporating user elements to enhance relevance), (2) integration (text interactions with a larger intervention platform), (3) tailoring (individualized feedback adaptive to participant needs), and (4) message intensity (message delivery frequency) [12]. All these features were observed in this study although not coded and quantified in accordance with the 4 principles above. The texts were generated autonomously by coaches in positively intended communications [10].

Other ICBT studies have been aimed at testing the efficacy of text messages in multimodal programming. In 1 RCT, an 8-week ICBT intervention was assessed, based on a web-based platform with text or video modules, self-reflection tasks, and homework assignments [3]. Weekly, text-chat sessions between participant and therapist focused on encouragement, problem identification, cognitive review, and program homework assistance. Mostly female adolescents (67/70, 96%; mean age 17.5 years) who reported depressive episodes (53/70, 76%) and had no prior treatment (45/70, 64%) were participants. ICBT and web-based text-chat session participants demonstrated a significant decrease in depression symptoms compared with minimal attention controls (d=0.86, 95% CI 0.37-1.35; *P*<.001).

In our RCT, alliance building disclosures were self-revealing texts that, when shared with coaches, seemed intended to increase trust and sensitive caretaking. The disclosure of upsetting events often evolved into empathic exchanges of reported distress. The discrimination of alliance building disclosures as identifiable communication patterns aligns with decades of theory and research on disclosure in association with stress resilience and future health [34,35]. Many mental health treatments, (especially psychodynamic treatments), center on disclosure and self-observation of inner experiences [36], and multiple studies support disclosure guided by clinicians who theoretically view self-disclosure as contributing to healing [37-41]. Our clinical observations support the importance of self-disclosures during NC-participant communications. However, in the previous research cited, there were minimal specifications of types and frequency of disclosures. Text messages provide the opportunity for both specification and quantification.

Several study limitations indicate interpretive cautions. Although 2 experienced qualitative analysts (CW and LPM) were responsible for defining text messaging categories and their definitions were confirmed by 2 other investigators (Nazanin Babaei and PR), other investigators might have reached differing qualitative judgments. The instrumentally definitive codes can be further verified in future analyses as the study-associated analyzable database contains all the texts exchanged between each participant-coach dyad during the 24-week intervention. Second, we focused on the texts sent by participants and not on the coach-sent texts proximal to participant texts. Nonetheless, what was confirmed was that self-disclosure communications (from participants to therapists) can regularly occur via text message with possible benefits. Third, we confronted sample size limitations due to slower-than-expected accrual in the primary randomized trial. This led to relatively wide CIs in our effect size estimates. A future, larger, study [42] will further assess our findings in this paper.

In the future, significant benefits can occur with text messaging use, a familiar communication format compatible with ICBT programming. While it has the advantage of tapping into comfortable and familiar communications, judgments on the optimal use of text messaging await further investigations. On the path to optimizing value is the differentiation of which types of sent texts are most closely associated with benefits. This was our study goal. Beyond the current findings, the methods developed and applicable can be implemented in future studies. Meanwhile, we will be integrating the apparent lessons from this study in our current trial [42].

## Abbreviations

BAI: Beck Anxiety Inventory
BDI-II: Beck Depression Inventory-II
BPI: Brief Pain Inventory
CBT: cognitive behavioral therapy
DSM-5: diagnostic and statistical manual of mental disorders
ICBT: internet-based cognitive behavioral therapy
MDD: major depressive disorder
NC: Navigator-Coach
RCT: randomized control trial
TAU: treatment as usual

## Data Availability

The data sets generated and analyzed during this study are available from the corresponding authors upon reasonable request.
